# Antibody targeting TSPAN12/β-catenin signaling in vasoproliferative retinopathy

**DOI:** 10.18632/oncotarget.23401

**Published:** 2017-12-18

**Authors:** Felicitas Bucher, Martin Friedlander, Kyungmoo Yea

**Affiliations:** Department of New Biology, Daegu Gyeongbuk Institute of Science and Technology, Daegu, Republic of Korea

**Keywords:** Tetraspanin12, antibody, retinopathy, VEGF

Abnormal vessel growth and breakdown of the blood retinal barrier are major causes of vision loss in western countries [1]. Over the past decade, we have seen an increasingly successful use of biological agents targeting vascular endothelial growth factor (VEGF) to treat diabetic retinopathy and age-related macular degeneration via intravitreal injections. However, long-term studies have shown that visual acuity still drops in over 30% of treated patients, while certain patient populations do not responds to anti-VEGF treatment at all [2, 3]. Discoveries of novel complementary targets are thus necessary to improve the therapeutic success. In our recent paper, we identified tetraspanin 12 (TSPAN12) as novel therapeutic target for retinal vascular disease, and developed a TSPAN12-targeting therapeutic antibody [4].

TSPAN12 belongs to the Tetraspanin family, which mainly includes cell surface proteins characterized by four transmembrane domains and two extracellular loops [5]. Tetraspanins interact with various cell surface proteins and regulate their intracellular trafficking, lateral diffusion and clustering at the plasma membrane [6]. They have also been associated with several pathological conditions such as tumor progression and metastasis [7]. In the retina, TSPAN12 is selectively expressed in the retinal vasculature and acts as a key regulator for retinal vascular development by activating β-catenin signaling. In 2009, Junge and colleagues first published results from a large-scale genetic screen suggesting that mutations in the previously uncharacterized TSPAN12 gene caused retinal vascular defects similar to those observed in FZD4, LRP5, and Norrin, which induced the blindness-causing disease familial exudative vitreoretinopathy [8]. With the help of TSPAN12-deficient mice, they proved TSPAN12 to be critical for the development of the retinal vasculature through the activation of β-catenin signaling. In vitro studies showed that TSPAN12 promotes the complex formation of Frizzled-4 (FZD4) and its co-receptor, low-density lipoprotein receptor-related protein 5 (LRP5).

While past studies focused on the role of TSPAN12 in retinal development, we are the first to show that TSPAN12/β-catenin signaling plays an important role in retinal neovascular disease [4]. In our present study, we developed anti-TSPAN12 antibodies and tested to see if they can reduce FZD4/TSPAN12-mediated β-catenin signaling and thus be used as a treatment for vasoproliferative retinopathy. For the selection of a high affinity antibody, a 48–amino acid peptide antigen encompassing the big extracellular loop of TSPAN12 was designed. Then, a phage library consisting of approximately 10^9^ human combinatorial antibodies was panned against the antigen. The selected TSPAN12 antibody was found to have a significant inhibitory effect on human umbilical vein endothelial cell functions such as migration and cell-cell adhesion. In addition, β-catenin expression was significantly decreased by the TSPAN12 antibody, suggesting an inhibition of TSPAN12/β-catenin signaling. The mechanism appears to involve the disruption of TSPAN12 interaction with FZD4, as was measured as a reduction during a co-immunoprecipitation experiment.

To investigate the therapeutic potential of this TSPAN12 antibody, we used two mouse models of vasoproliferative retinopathy, the oxygen-induced retinopathy (OIR) model and the very-low-density lipoprotein receptor (VLDLR) knockout model [4]. In both models, intravitreal injection of the TSPAN12 antibody significantly reduced abnormal vessel growth. The TSPAN12 Ab selectively targeted β-catenin signaling in vascular endothelial cells *in vivo* without affecting retinal VEGF levels. Combination therapy with a known anti-VEGF agent such as Aflibercept demonstrated significant therapeutic synergy providing the opportunity to decrease therapeutic doses of anti-VEGF agents and reduce unwanted side-effects. These aspects support the potential clinical use of the TSPAN12 antibody.

Due to the selective expression of TSPAN12 in retinal vascular endothelial cells the TSPAN12 antibody may also serve as delivery vehicle for endothelial specific therapeutics. It may be possible to develop a novel fusion protein similar to Aflibercept that combines the TSPAN12 and VEGF-receptor antigen domain. Furthermore in the OIR model, the TSPAN12 Ab more strongly supported physiologic revascularization into hypoxic retinal tissue compared to anti-VEGF treatment. Therefore, modulating TSPAN12 activity may also present a promising target to encourage physiologic revascularization of avascular areas in diabetic retinopathy or vein occlusion, a yet unsolved problem.
