# Correction: Antibody targeting TSPAN12/β-catenin signaling in vasoproliferative retinopathy

**DOI:** 10.18632/oncotarget.26102

**Published:** 2018-09-04

**Authors:** Felicitas Bucher, Martin Friedlander, Kyungmoo Yea

**Affiliations:** Department of New Biology, Daegu-Gyeongbuk Institute of Science and Technology (DGIST), Daegu, Republic of Korea

**This article has been corrected:** The correct affiliation for Kyungmoo Yea is given below:

Department of New Biology, Daegu-Gyeongbuk Institute of Science and Technology (DGIST), Daegu, Republic of Korea

Original article: Oncotarget. 2018; 9:12538-12539. https://doi.org/10.18632/oncotarget.23401

